# Neurodegeneration in type 2 diabetes: Alzheimer's as a case study

**DOI:** 10.1002/brb3.1577

**Published:** 2020-03-14

**Authors:** Jalaja Madhusudhanan, Gowthaman Suresh, Vasudharani Devanathan

**Affiliations:** ^1^ Department of Biology Indian Institute of Science Education and Research (IISER) Tirupati Tirupati India

**Keywords:** Alzheimer's disease, neurodegeneration, type 2 diabetes mellitus, type 3 diabetes

## Abstract

**Introduction:**

Rigorous research in the last few years has shown that in addition to the classical mechanism of neurodegeneration, certain unconventional mechanisms may also lead to neurodegenerative disease. One of them is a widely studied metabolic disorder: type 2 diabetes mellitus (T2DM). We now have a clear understanding of glucose‐mediated neurodegeneration, mostly from studies in Alzheimer's disease (AD) models. AD is recognized to be significantly associated with hyperglycemia, even earning the term “type 3 diabetes.” Here, we review first the pathophysiology of AD, both from the perspective of classical protein accumulation, as well as the newer T2DM‐dependent mechanisms supported by findings from patients with T2DM. Secondly, we review the different pathways through which neurodegeneration is aggravated in hyperglycemic conditions taking AD as a case study. Finally, some of the current advances in AD management as a result of recent research developments in metabolic disorders‐driven neurodegeneration are also discussed.

**Methods:**

Relevant literatures found from PubMed search were reviewed.

**Results:**

Apart from the known causes of AD, type 2 diabetes opens a new window to the AD pathology in several ways. It is a bidirectional interaction, of which, the molecular and signaling mechanisms are recently studied. This is our attempt to connect all of them to draw a complete mechanistic explanation for the neurodegeneration in T2DM. Refer to Figure 3.

**Conclusion:**

The perspective of AD as a classical neurodegenerative disease is changing, and it is now being looked at from a zoomed‐out perspective. The correlation between T2DM and AD is something observed and studied extensively. It is promising to know that there are certain advances in AD management following these studies.

## Graphical abstract

We have highlighted the role of hyperglycemia in neurodegenerative diseases such as Alzheimer's disease. In the last 1 or 3 decades, this has become very prominent and it is interesting to review the same.

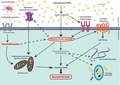

## 1 INTRODUCTION

Neurodegenerative diseases are generally characterized by cellular accumulation of misfolded proteins, ROS production due to mitochondrial dysfunction, and disruption of the autophagy machinery in neuronal cells. Alzheimer's disease (AD) despite being a neurodegenerative disease with a well‐explored disease pathology is still of much interest to researchers. Conventional mechanisms of neurodegeneration in patients with AD include beta‐amyloid (Aβ) plaque accumulation and tau protein neurofibrillary tangles formation in the brain, eventually leading to dementia and other behavioral problems, and ultimately to death. For a long time, these mechanisms were the focus of AD research, leading to a growing hope that effective drugs against AD would be discovered soon. Now, we understand that the changes leading to AD are also aging‐related, genetic and inheritable, and thus are not easily reversible. Recent progress in AD research has demonstrated that there are several other external factors widely causing the emergence of AD pathologies, such as obesity, diabetes, brain injury, neurotoxicity, and infections (Dosunmu, Wu, Basha, & Zawia, 2007; Pugazhenthi, Qin, & Reddy, 2017). Although it has been more than a decade since the idea of diabetes mellitus as a causal disorder of many neuronal diseases originated, this link has been less explored (Seaquist, 2010). This oversight is likely due to inadequate methodologies and lack of appropriate testable models. Recently, this idea has gained momentum since increased AD pathology has been observed in AD patients with T2DM (Mehla, Chauhan, & Chauhan, 2014). These unconventional modes of AD emergence are likely to be lifestyle‐related and may be controlled and even reversed if correctly targeted. Therefore, it is essential to focus research on the relationship between metabolic disorders and neuronal alterations resulting from such disorders, in order to unravel the molecular mechanisms behind it (Calvo‐Ochoa & Arias, 2015). Here, we provide a concise review of diabetes‐associated mechanisms of neurodegeneration and cognitive impairment, with an emphasis on the pathophysiology of AD.

## 2 PATHOPHYSIOLOGY OF AD

### 2.1 Classical AD pathology

Alzheimer's disease has been recognized as a deadly neurological disease since its discovery at the beginning of the 20th century by Dr. Alois Alzheimer and continues to be a significant neurodegenerative disease without a cure. It is a prominent cause of dementia in elderly people all over the world. According to the World Alzheimer Report 2015, there are approximately 46.8 million people worldwide diagnosed with dementia. In AD, irreversible neurodegeneration causes severe damage to the brain tissue and a reduction in size of the brain (Bernardes et al., 2017; Hannah, 1936). The term neurodegeneration refers to the progressive death of neurons due to multiple causes, some of which are widely explored as in the case of AD. Classically, AD is characterized by the accumulation of protein both intracellularly and extracellularly. The main culprit is the hydrophobic beta‐amyloid (Aβ) peptide, secreted in the extracellular space after the proteolytic cleavage of a transmembrane glycoprotein amyloid precursor protein (APP) by beta‐secretase followed by gamma‐secretase enzymes (O’Brien & Wong, 2011). APP is a transmembrane protein and an integral part of synapses in the brain, while the soluble form of Aβ has a crucial role in neuronal growth and survival in physiological conditions (O’Brien & Wong, 2011; Pearson & Peers, 2006). However, an imbalance in production and/or degradation of insoluble Aβ peptides 40–42 amino acids in length and 4.2 kDa in size leads to its accumulation and polymerization, creating plaques that are detrimental to the cell (O’Brien & Wong, 2011). Another conventional mechanism of neurodegeneration in AD is the neurofibrillary tangles formed by aggregation of tau protein in the cytoplasm due to its misfolding after hyperphosphorylation. The normal physiological function of tau protein is to help stabilize the neuronal cytoskeleton (Mietelska‐Porowska, Wasik, Goras, Filipek, & Niewiadomska, 2014). In patients with AD, aggregates of tau protein do not undergo degradation by autophagy, a protein degradation machinery, leading to continuous accumulation of this protein (Iqbal, Liu, Gong, Alonso, & Grundke‐Iqbal, 2009). The deposition of tau eventually causes oxidative stress to the cell, and the production of reactive oxygen species (ROS) by the mitochondria results in the activation of apoptotic signals, leading to enhanced neuronal cell death (Iqbal et al., 2009; Liu et al., 2015). Since mature neurons cannot regenerate, degeneration of neurons leads to loss of connections between neurons which are crucial for memory retention. As a result, older adults with AD show various symptoms of dementia, such as confusion, difficulty in thinking, recognizing people, writing, speaking, and reading, as well as other behavioral problems (Ropper, 1979).

Currently, researchers are working on various aspects of the disease to elucidate complete molecular mechanisms, identify drug targets, and design early diagnostic tools and to plan effective AD management methods. Recent clinical studies have demonstrated a dramatic correlation between AD and metabolic diseases such as type 2 diabetes mellitus (ClinicalTrials.gov Identifiers: NCT02501876, NCT02360527, NCT03578991). Hence, AD is now recapturing the attention of neuroscientists as a possible complication of defective glucose metabolism (Bianchi & Manco, 2018).

### 2.2 Diabetes: A new window to AD pathology

**Figure 1:**
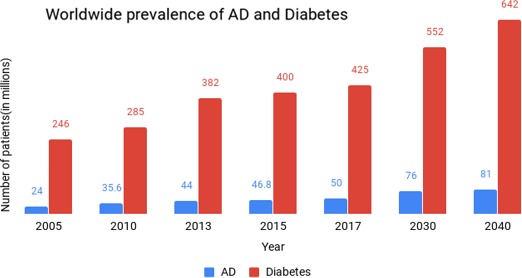
Graphical representation of the estimated number of AD and Diabetes patients worldwide. It shows a steady increase in the number of AD patients along with the huge increase in diabetic patients every year, indicating a strong correlation between them. The numbers for 2030 and 2040 are extrapolated from the current statistics. (Sources: IDF (International Diabetes Federation), ADI (Alzheimer's Disease International) and WHO (World Health Organization). Data points are the estimates reported in the websites of these organizations, and they are compiled and represented as a histogram for comparison)

Diabetes mellitus is a lifestyle disease prevalent among people from all over the globe. It is a condition in which the glucose metabolism of the body is dysregulated, resulting in a high level of glucose in the blood. According to a recent report by International Diabetes Federation (IDF), the number of patients with diabetes in the world has increased from 108 million in 1980 to 425 million in 2017, indicating that every 11th person in the world is diabetic (Risk Factor Collaboration, 2016). These numbers probably underestimate the actual number of patients with diabetes, since one out of two people remains undiagnosed in most developing countries. According to estimates by the World Health Organization (WHO), by 2030 developing countries like India will contribute five times more than developed countries to the prevalence of diabetes and diabetes‐related deaths (Wild, Roglic, Green, Sicree, & King, 2004) (Figure 1). This could also be an indication of the alarming number of patients with AD in developing and underdeveloped countries, where unavailability of modern diagnostic techniques and new treatment strategies for AD are contributing to a major health crisis (Kalaria et al., 2008).

The cause for increased blood glucose levels in a patients with diabetes can vary based on which diabetes is classified into two major types: type 1 and 2 diabetes. Type 2 diabetes is the more common type. Type 2 diabetes mellitus (T2DM) can be due to insufficient insulin production by the pancreatic beta cells, or due to insulin resistance in the body. Insulin resistance is the inability of a cell to respond adequately to insulin signaling, resulting in decreased glucose uptake by the cell (Saini, 2010). Consequently, insulin‐resistant cells die leading to severe complications and inefficient functioning of multiple organs. Diabetic stroke, hypertension, cardiovascular disease, kidney failure, and liver damage are some of them. Neurodegeneration has been recently added to this list (Harding, Pavkov, Magliano, Shaw, & Gregg, 2019). Notably, there are numerous studies on the association between neurodegenerative disease AD and T2DM (Jayaraman & Pike, 2014). Although such studies repeatedly emphasize the relation between AD and T2DM, it is important to note that diabetes‐related risk factors are not sufficient to cause AD. However, they indeed promote AD pathology by triggering neurodegeneration by various mechanisms (Moroz, Tong, Longato, Xu, & De La Monte, 2008). In severe cases of T2DM, glucose toxicity in the brain is mainly due to oxidative stress triggered by amplified free radical formation and decreased free radical scavenging mechanisms (Tomlinson & Gardiner, 2008). Excessive glucose levels in the neuronal niche can cause lipid peroxidation, carbonylation of proteins, and DNA damage which causes irreparable harm to neurons (Ito, Sono, & Ito, 2019). High amounts of free radical production are associated with inflammation. As a consequence of inflammatory pathway activation, metalloproteinases may damage blood–brain barrier (BBB) integrity and cause brain edema (Kamada, Yu, Nito, & Chan, 2007). Hyperglycemic conditions in the brain promote accumulation of lactic acid, which leads to intracellular acidosis and sequentially incites mitochondrial dysfunction and energy failure (Anderson, Tan, Martin, & Meyer, 1999).

Correlations between the pathology of AD and T2DM have been observed. Various studies using AD animal models have shown that diet‐induced insulin resistance/chemically induced insulin signaling impairment increases AD pathology (Hascup et al., 2019; Mehla et al., 2014). This implies that insulin resistance in a patients with diabetes may lead to problems related to memory and cognition. There is also evidence to support the idea that patients with diabetes are more susceptible to develop AD (Cheng, Huang, Deng, & Wang, 2012). Researchers have postulated that diabetes could be a novel mechanism of neurodegeneration wherein the classical AD pathophysiology can be explained from the perspective of unregulated insulin/IGF (insulin‐like growth factor) signaling and improper glucose metabolism. The diabetic brain starves because of insufficient expression of glucose transporters (mainly GLUT4) on the membrane of neurons without which glucose cannot be transported into the cells (Blázquez, Velázquez, Hurtado‐Carneiro, & Ruiz‐Albusac, 2014). This can result in oxidative stress in the mitochondria, causing neurons to degenerate by induction of apoptosis (Sripetchwandee, Chattipakorn, & Chattipakorn, 2018). On the other hand, impaired insulin/IGF signaling in the brain is also implied in hyperphosphorylation of tau protein by one of the many kinases (PI3K/Akt/MAPK) in the downstream of insulin signaling pathways. Disrupted regulation of any of these kinases in the diabetic brain can lead to tau hyperphosphorylation and accumulation, one of the hallmarks of AD (de la Monte, 2014; Plum, Schubert, & Brüning, 2005). After several clinical studies, the association between T2DM and AD has finally become well‐established, although the molecular mechanism remains to be unveiled (Plum et al., 2005). A point to be noted regarding studies using AD animal models is that patients with AD present with metabolic disorders and many symptoms of diabetes, which may not be fully represented by animal models (Chakrabarti et al., 2015). Close analysis and further studies are necessary to understand the possible bidirectional mechanism involved in mutually promoting AD and T2DM pathologies (Shinohara & Sato, 2017).

## 3 MECHANISMS OF T2DM‐DERIVED NEURODEGENERATION IN AD

### 3.1 Neurodegenerative role of Amylin

**Figure 2:**
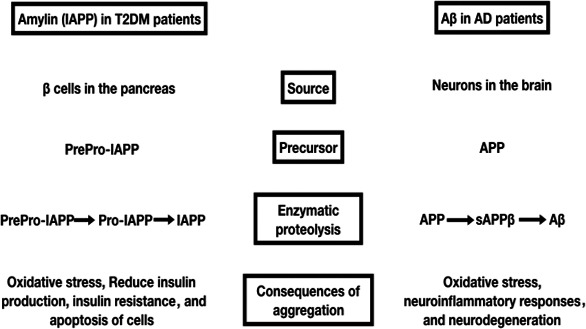
Comparison between the role of amylin (IAPP) in T2DM patients and Aβ (beta‐amyloid) in AD patients. It supports the fact that the dysfunction and accumulation of Amyloidogenic peptides are common causes for both the pathologies. Amylin is now considered as one of the prominent links in the molecular mechanism of glucose‐mediated neurodegeneration

“Islet amyloid polypeptide” (IAPP) or “amylin” is a less understood peptide hormone synthesized and cosecreted along with insulin by the pancreatic β cells (Despa & DeCarli, 2013). It is produced in minute quantities when compared to insulin, but functions similar to insulin. Amylin has recently become a topic of focus in current AD research (Mietlicki‐Baase, 2016). Amylin is structurally very similar to beta‐amyloid (Lim et al., 2010). Like Aβ, amylin is also processed through multiple steps by proteolytic enzymes to finally form amylin or islet amyloid polypeptide (IAPP) (Akter et al., 2016; Nagamatsu, Nishi, & Steiner, 1991; Sanke, Bell, Sample, Rubenstein, & Steiner, 1988). Importantly, amylin aggregates have been noticed in the pancreatic islets of patients with T2DM (Mietlicki‐Baase, 2016; Mitsukawa et al., 1990). It leads to apoptosis of β cells and thus a reduction in insulin production (Lutz & Meyer, 2015). Amylin deposition also contributes to insulin resistance and oxidative stress responses observed in these cells (Lutz & Meyer, 2015). Interestingly, amylin can also cross the BBB and amylin receptors are distributed in some parts of the CNS, as observed in the case of insulin and its receptors (Mietlicki‐Baase & Hayes, 2014) (Figure 2). However, amylin accumulates in several peripheral organs of patients with T2DM as well, which explains the hyperamylinic condition in the diabetic brain (Jackson et al., 2013). Hyperamylinemia eventually leads to deleterious effects in the brain and enhances the symptoms of AD pathology (Lim, Ittner, Lim, & Götz, 2008). Thus, amylin is considered a pancreas‐derived neuropeptide playing a crucial role in the development of AD pathology in patients with T2DM (Jackson et al., 2013). Although the actual mechanism of amylin‐mediated neurodegeneration is not completely known, attempts have been made to develop our understanding. One such study involves AD patients with T2DM and diabetic HIP (human islet amyloid polypeptide) rats (a new model for T2DM in which rats express human amylin in pancreatic islets). The study revealed that the accumulation of amylin‐Aβ complex in the brain of AD patients with T2DM activates the production of cytokines such as IL‐1β, which in turn enhances neuroinflammatory immune responses leading to gradual degeneration of neurons (Verma et al., 2016). Amylin and its analogs are shown to interact and activate different downstream molecules in the insulin signaling pathway (Moon, Chamberland, & Mantzoros, 2012; Nassar, Badae, & Issa, 2018).

Parallel research exploiting the structural and biophysical similarities between amylin and beta‐amyloid peptide has unearthed another fascinating finding that patients with AD significantly overexpress amylin receptors (Jhamandas et al., 2011). It is already known that Aβ and amylin can bind to the same receptor, which indicates a probable amylin receptor‐mediated Aβ action in patients with AD (Nassar et al., 2018). In vitro studies have shown that blocking amylin receptors could mitigate the electrophysiological effects of Aβ and confer neuroprotection (Jhamandas et al., 2011). These studies provide the rationale for considering amylin receptors as a reliable novel therapeutic target for the treatment of AD.

### 3.2 Impaired insulin signaling in beta‐Amyloid plaque formation and tau hyperphosphorylation

Insulin signaling is vital for several functions of the brain. Some of these include synaptogenesis, plasticity, neuroregeneration, learning, memory, neuritogenesis, and repair (Tumminia, Vinciguerra, Parisi, & Frittitta, 2018). Insulin also regulates APP metabolism in neurons (Plum et al., 2005; Tumminia et al., 2018). Hence, an imbalance in insulin signaling can reflect on the metabolism and processing of APP, which eventually leads to the accumulation of Aβ in the cell—a major cause for neurodegeneration in AD. As evidence for the potential role of insulin signaling in neurodegeneration in AD, significantly reduced expression of insulin receptor (IR) has been observed in the brains of patients with AD (Frazier et al., 2019). Decreasing the intracellular accumulation of Aβ by modulating insulin signaling is another strong indication of the vital role of insulin signaling in AD pathology (Craft, 2006). Interestingly, APP appears to be essential for maintaining healthy glycemic regulation. Studies using APP knockdown mice demonstrate that these mice develop metabolic disorders as well (Kulas et al., 2018).

Furthermore, hyperphosphorylation of the tau protein, one of the critical features of AD pathology is also increased due to impaired insulin signaling in the brain of patients with T2DM (Plum et al., 2005; Tumminia et al., 2018). Glycogen synthase kinase‐3 (GSK‐3) is an enzyme downstream to IR in the insulin signaling cascade, and its GS phosphorylating activity is downregulated by insulin. GSK‐3 has been recently demonstrated to phosphorylate tau proteins. Thus, altered insulin signaling may modulate GSK‐3β activity, leading to the hyperphosphorylated state of tau proteins observed in the brains of patients with T2DM (Frazier et al., 2019). The hyperphosphorylated tau proteins eventually get converted into neurofibrillary tangles, which is one of the key indications of neurodegeneration in AD.

### 3.3 Neuroinflammation and defective insulin signaling

**Figure 3:**
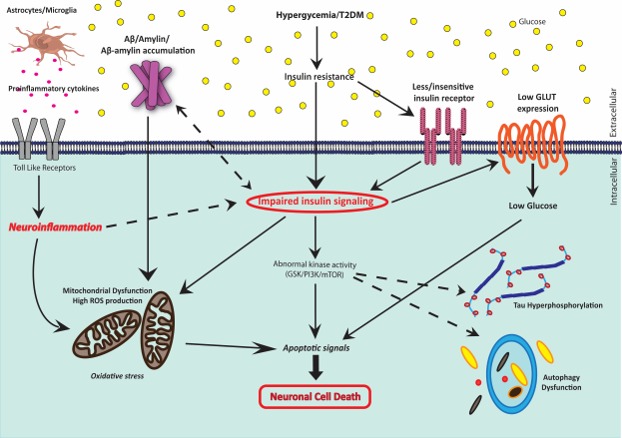
Schematic representation of the different mechanisms involved in glucose‐mediated neurodegeneration. Impaired insulin signaling is at the center in T2DM patients, which leads to various AD symptoms and then ultimately to the neuronal cell death

It is well‐known that neuroinflammatory pathways can cause deleterious effects on neuronal cells. In the hyperglycemic condition, neuroinflammatory pathways can be induced in numerous ways (Refer to Figure 3). First, increased mitochondrial activity creates a stressful environment within the cell, thus enhancing ROS production which leads to the activation of inflammatory pathways. One of the other key features of T2DM is the overproduction of proinflammatory cytokines such as TNF‐α and IL‐6, in part due to hyperactivation of microglia and astrocytes, the immune cells of the brain (Bahniwal, Little, & Klegeris, 2017; Nasoohi, Parveen, & Ishrat, 2018). Persisting inflammation and abnormal levels of circulating cytokines that may even breach the BBB can be observed in patients with T2DM (Nasoohi et al., 2018). TNF‐α promotes various stress‐sensitive kinases which induce serine phosphorylation of IRS‐1, an essential molecule in the insulin signaling cascade which is usually activated by phosphorylation at a tyrosine residue to propagate the insulin signal (Nasoohi et al., 2018). Thus, increased cytokine levels in the brain can lead to defective insulin signaling, which is one of the mechanisms through which T2DM affects brain functions (Ferreira, Clarke, Bomfim, & Felice, 2014). It is clear that T2DM‐induced chronic inflammation has a significant impact on the brain and is one of the important causal mechanisms of many neurological disorders such as AD and multiple sclerosis (MS) (Van Dyken & Lacoste, 2018). High levels of TNF‐α in the cerebrospinal fluid (CSF) of patients with AD indicate that inflammation‐induced impaired brain insulin signaling may be a major cause of insulin resistance observed in these patients. This implies that in patients with AD, impaired cerebral insulin signaling due to neuroinflammation may be a possible link between cerebral dysfunction and T2DM (Ferreira et al., 2014; Mehla et al., 2014; Nasoohi et al., 2018). Another mechanism by which hyperglycemia‐induced neuroinflammatory pathways may affect the brain is through Toll‐like receptor 4 (TLR4). TLR4 is highly expressed in all parts of the CNS and may be one more link between T2DM and AD (Huang, Jin, Zhou, Shi, & Jin, 2017). TLR4 signaling pathways are continuously active in diabetes, leading to insulin resistance. Although activation of TLR4 in the initial stages of AD helps remove Aβ depositions, long‐term activation appears to be detrimental to the brain. Chronic TLR4 activation causes chronic inflammation, which leads to diabetic neuropathy and AD (Huang et al., 2017).

### 3.4 Cognitive impairment in T2DM

The CNS is one of the most important targets of insulin. Insulin receptors (IRs) are widely expressed in different parts of the brain, especially in the hippocampus. Insulin mediates metabolic homeostasis and regulates neurotrophic processes and synaptic plasticity of the brain (Calvo‐Ochoa & Arias, 2015; Gudala, Bansal, Schifano, & Bhansali, 2013; Nguyen et al., 2018; Zhao, Chen, Quon, & Alkon, 2004). Earlier, in vitro studies using hippocampal cell cultures established a neuroprotective role for insulin (Duarte, Proença, Oliveira, Santos, & Rego, 2006; Stockhorst, Fries, Steingrueber, & Scherbaum, 2004). As mentioned before, insulin can activate PI3‐K/Akt and S6K/mTOR kinase pathways which apart from regulating glucose metabolism also have a pivotal role in neuronal growth and synaptic plasticity (Calvo‐Ochoa & Arias, 2015; Stockhorst et al., 2004; Zhao et al., 2004). Importantly, it has been observed that insulin can mediate the expression and recruitment of AMPA, NMDA, and GABA receptors in the postsynaptic cluster and control release of neurotransmitters such as acetylcholine and norepinephrine, all of which are directly related to the generation of long‐term potentiation (LTP) needed for long‐term memory in the hippocampus (Boyd, Clarke, Muther, & Raizada, 1985; Skeberdis, Lan, Zheng, Zukin, & Bennett, 2002; Van Der Heide, Kamal, Artola, Gispen, & Ramakers, 2005; Wan et al., 1997). Several transgenic and genetic T2DM models reported significant reduction in LTP, synaptic damage, decreased expression of neurotrophic factors, compromised BBB integrity, and neuroinflammation. These processes are associated with hippocampus‐based cognitive impairment and memory deficit (Calvo‐Ochoa & Arias, 2015).

Clinical findings and numerous epidemiological studies also support the beneficial role of insulin on cognition, and now, it is well‐established that the insulin resistance associated with T2DM can also lead to vascular dementia (Skeberdis et al., 2002). Vascular dementia is a general term for the cognitive impairment associated with any metabolic disorder; in particular, diabetes‐induced cognitive impairment is known as diabetic encephalopathy (DE). Furthermore, all these symptoms may eventually lead to the onset of AD. Evidently, all AD pathophysiologies are well linked to insulin signaling and T2DM (Johnson, Torres, Impey, Stevens, & Raber, 2017). These studies indeed indicate the importance of insulin in cognitive functions, learning, and memory formation. Hence, when compared to nondiabetic individuals, patients with diabetes are at approximately 70% higher risk for the development of vascular dementia or AD (Gudala et al., 2013).

### 3.5 Autophagy dysfunction and diabetes‐induced AD

Autophagy is the catabolic degradation of misfolded or nonfunctional proteins and parts of damaged organelles (Calvo‐Ochoa & Arias, 2015; Chen et al., 2019). Autophagy dysfunction is known to contribute to several neurodegenerative diseases, including AD (Calvo‐Ochoa & Arias, 2015; Kiriyama & Nochi, 2015; Komatsu et al., 2006). Aβ accumulation and neurofibrillary tangle formation may be caused by the downregulation of autophagy in neuronal cells. Recently, it has been demonstrated that downregulation of autophagy is also associated with T2DM, which indirectly suggests that autophagy dysfunction could be another important mechanism by which it disrupts the homeostasis of neuronal cells and induces neurodegeneration (Kanamori et al., 2015; Wilson, Magnaudeix, Yardin, & Terro, 2014). The PI3K/mTOR pathway has an essential role in the regulation of autophagy, which is disrupted in conditions such as insulin resistance and/or impaired insulin signaling (Calvo‐Ochoa & Arias, 2015). Researchers are also interested in finding new therapeutic targets using knowledge of the shared mechanisms of disease pathology between AD and T2DM. Pharmacological autophagy induction could be a viable therapeutic strategy not only for AD but for many other neurodegenerative diseases (Chen et al., 2019; Friedman, Qureshi, & Yu, 2015).

### 3.6 Involvement of cell adhesion molecules in glucose transport and AD

Prion protein (PrPc) is a neuronal membrane protein involved in cell–cell adhesion and intercellular communication. Prion has been shown to interact with beta‐amyloid and is thus suspected to have a significant role in AD‐related pathologies (Jarosz‐Griffiths, Noble, Rushworth, & Hooper, 2016). Interestingly, prion antagonists are currently being used effectively to reduce the neurotoxic effects of the prion‐β‐amyloid interaction and cognitive deficit in AD models (Sagare, Sweeney, Nelson, Zhao, & Zlokovic, 2019). Recently, correlations have been observed between prion protein (PrPc) and its modulatory effect on intracellular iron levels in various cell types including neuronal cells. Intracellular iron overload is considered a risk factor for diabetes. Imbalance in iron homeostasis in diabetes results in beta cell failure and insulin resistance, which are the hallmarks of T2DM (Simcox & McClain, 2013). The underlying molecular mechanism is not clear, but it has been observed that iron overload in diabetes induces hypoxia and ROS production, which leads to beta cell damage and decreased insulin gene expression (Kaneto, Katakami, Matsuhisa, & Matsuoka, 2010; Walter et al., 2002). Also, hypoxia‐inducible factors (HIF) may downregulate GLUT1 and GLUT2 expression, thus enhancing hypoglycemic conditions in the cell. In brief, apart from oxidative stress due to HIF and ROS, iron overload may induce glucose‐mediated neurodegeneration (Merelli et al., 2018). A role for prion in glucose uptake of the cell by altering the GLUT2 expression has also been reported, which indicates that prion can indirectly affect glucose metabolism (Ashok & Singh, 2018).

## 4 CURRENT RESEARCH ADVANCES AND AD MANAGEMENT

Both AD and T2DM are called amyloidoses because of the overlapping mechanisms noted between them, as discussed above. Currently, very few FDA approved drugs are available for the treatment of AD, and these are only partially effective in preventing further deterioration of the condition. Therefore, at present, the main goals of AD researchers are to find new drugs for designing better treatment strategies and to investigate newer therapeutic targets to reverse AD pathology. Brain insulin resistance and reduced glucose uptake by neuronal cells due to ineffective insulin signaling are some of the common pathophysiological mechanisms observed in all neurodegenerative processes, and hence, novel research advances in this direction will be widely appreciated (Griffith, Eid, Rose, & Patrylo, 2018; Kim & Feldman, 2015).

### 4.1 Insulin sensitizers for AD treatment

Soon after the association between neurodegeneration and T2DM was established, the idea of using insulin as a therapy for neurodegenerative diseases emerged (Kim & Feldman, 2015). Thus, insulin therapy was developed to improve cognition and delay the onset of memory loss and confusion in patients with AD (Chapman, Schiöth, Grillo, & Benedict, 2018). In most cases of patients with T2DM, insulin signaling is impaired or the cell is insensitive to insulin (insulin resistance). To treat this, insulin sensitizers are used (Ye, Luo, Xiao, Yu, & Yi, 2016). Some of the commercially available, famous insulin sensitizers are Metformin and Thiazolidinediones (TZDs). Interestingly, these insulin sensitizers have shown therapeutic potential against AD, which soon came to be known as type 3 diabetes (De La Monte & Wands, 2008; Moreira, Campos, & Soldera, 2013). In order to analyze the effects of commercially available insulin sensitizers on AD, researchers have used them to restore glucose metabolism in the brain of AD mouse models and demonstrated that these drugs are effective in overcoming AD symptoms by attenuating neuroinflammation and tau hyperphosphorylation (Yu et al., 2015). Metformin is one of the well‐known drugs for maintaining glycemic control in patients with T2DM. It was recently reported as a potential therapeutic for AD as it improves cognitive performance and decreases the chances of developing diabetic encephalopathy (Hsu, Wahlqvist, Lee, & Tsai, 2011; Ng et al., 2014; De Oliveira et al., 2016; Pintana, Apaijai, Pratchayasakul, Chattipakorn, & Chattipakorn, 2012). In vitro and in vivo studies using diabetic mouse models have reported that metformin treatment improves autophagic clearance of hyperphosphorylated tau protein in patients with AD (Chen et al., 2019). This is indeed a promising step toward the generation of effective therapeutics targeting neurodegeneration in both AD and T2DM‐associated early‐AD patients.

### 4.2 Neuroprotective role of Amylin

Amylin as discussed before is a relative new candidate in AD research. Currently, there are two controversial sides regarding the role of amylin in the brain. Apart from its neurodegenerative and neurotoxic effects, amylin has surprisingly shown some neuroregenerative and neuroprotective effects as well (Adler et al., 2014; Bharadwaj et al., 2017). Interestingly, when scientists investigated correlations between cognitive efficiency in patients with T2DM and amylin concentration, in the initial stages amylin deposition in the brain caused a detrimental effect, while in the later stages when the β cells failed, amylin had a beneficial role. It helped in Aβ autophagic clearance, and an improvement in cognition was also observed (Grizzanti, Corrigan, & Casadesus, 2018; Patrick et al., 2019). In addition, various functions of amylin in the CNS have been discovered; improvement of glucose metabolism (Roth, 2013), relaxation of cerebrovascular structures (Edvinsson, Goadsby, & Uddman, 2005), and enhancement of neural regeneration (Trevaskis et al., 2010). Currently, amylin and amylin analogs are considered as potential therapeutic candidates for diabetes as well as for cognitive improvement in patients with AD (Grizzanti, Lee, Camins, Pallas, & Casadesus, 2016; Wang et al., 2017).

### 4.3 Antidiabetic drugs and AD management

Although diabetes research is at its peak with several new antidiabetic drugs, advanced patient‐specific stem cell transplant/therapy, and insulin therapy, we are yet far from identifying a permanent cure for the “silent killer” (Abdelalim, Bonnefond, Bennaceur‐Griscelli, & Froguel, 2014). Patients with diabetes are also under the risk of early onset of dementia and other age‐related diseases such as AD. Recently, there have been many attempts to prevent the AD‐like alterations induced by inflammation and oxidative stress in patients with T2DM. While some of them target cognitive improvement of patients with AD using commercially available antidiabetic drugs/insulin sensitizers, others explore the beneficial effects of glucose metabolism‐related natural peptides or synthetic mimics/analogs of these natural peptides on neurodegeneration (Moreira et al., 2013). Glucagon‐like peptide 1 (GLP‐1), also known as incretin, is a hormone produced by enteroendocrine L cells that impacts food ingestion. It acts as a neuropeptide in the brain and also helps in glucose‐stimulated insulin secretion from pancreatic islets. GLP‐1 may regulate glucose metabolism and improve cognition, thus serving as a future treatment strategy for diabetes‐associated AD. GLP‐1 is generally considered to be neuroprotective and anti‐inflammatory, as it works by attenuating neuroinflammation (Qin, Chong, Rodriguez, & Pugazhenthi, 2016). Moreover, it is known to improve insulin sensitivity and promote neurogenesis (Bae & Song, 2017; Tai, Liu, Li, Li, & Hölscher, 2018). Fibroblast growth factor 21 (FGF21) also demonstrated similar effects on improvement of cognitive function after high fat and sugar diet (HFD)‐induced cognitive dysfunction in mice, probably due to its anti‐inflammatory properties (Wang et al., 2018). Interestingly, GLP‐1 receptor agonists commercially used for the treatment of T2DM, such as *lixisenatide* and *liraglutide,* have been tested on AD mouse models and shown to have neuroprotective effects. These drugs appear to reverse all the classical pathophysiologies of AD including strong LTP in the hippocampus, improving synaptic plasticity (Gault & Hölscher, 2008a), increasing number of synapses, and reduction in the Aβ accumulation and neuroinflammatory responses (McClean & Hölscher, 2014a, 2014b). Along similar lines, extensive studies have been performed using glucose‐dependent insulinotropic polypeptide (GIP), another less discussed peptide hormone which targets pancreatic islets enhancing beta cell growth and differentiation, and promoting insulin release (Gault, O’Harte, & Flatt, 2003). It also independently helps regulate blood glucose levels (Irwin et al., 2006) which makes it an attractive target for T2DM drugs. But because GIP is prone to degradation by proteases, it has a very short half‐life in the bloodstream (Gault & Hölscher, 2008b; Irwin et al., 2006). Thus, the newer approach is to develop long‐lasting GIP agonists to diminish the potential risk of cognitive impairment and neurodegeneration due to T2DM (Gault & Hölscher, 2008b). These recent developments toward designing an effective treatment strategy for neurodegenerative diseases have been summarized in Table 1.

**Table 1:** Antidiabetic drugs in clinical trials for neurodegenerative diseases

Drug	Type	Status	Data availability statement
Liraglutide	GLP‐1 analog	FDA approved drug for T2DM and in phase IIb clinical trial (NCT01843075) (Batista et al., 2018; Femminella et al., 2019) for AD	The data that support the findings of this study are openly available in PubMed at https://doi.org/10.1002/path.5056 (Batista et al., 2018)
Pioglitazone	Peroxisome proliferator‐activated receptor gamma (PPAR‐gamma) agonist, thiazolidinedione insulin sensitizer	FDA approved drug for T2DM and in phase II clinical trial for AD (NCT00982202) (Galimberti & Scarpini, 2017; Geldmacher, Fritsch, McClendon, & Landreth, 2011)	The data that support the findings of this study are openly available in PubMed at https://doi:10.1001/archneurol.2010.229 (Geldmacher et al., 2011)
Exendin‐4 (or Exenatide)	GLP‐1 agonist	FDA approved for T2DM and in phase II clinical trial for AD and (NCT02847403) Parkinson's disease (NCT01174810) (Aviles‐Olmos et al., 2013)	The data that support the findings of this study are openly available in PubMed at https://doi.org/10.1172/JCI68295 (Aviles‐Olmos et al., 2013)
Lixisenatide/Adlyxin	GLP‐1 receptor agonist	FDA approved drug for T2DM and in phase II clinical trial for PD (NCT03439943)	The data that support the findings of this study are openly available in Clinical Trials at https://clinicaltrials.gov/ct2/show/NCT03439943 (Study to Evaluate the Effect of Lixisenatide in Patient With Parkinson's Disease n.d.)
Metformin	Biguanide‐Insulin sensitizer	FDA approved drug for T2DM and in phase II clinical trial for AD (NCT00620191)	The data that support the findings of this study are openly available in PubMed at https://10.1212/01.wnl.0000140292.04932.87 (Luchsinger, Tang, Shea, & Mayeux, 2004)
Telmisartan	Telmisartan is an Angiotensin 2 receptor blocker	FDA approved drug for hypertension and in phase III clinical trial (NCT00274118) for T2DM and in phase III clinical trial for AD (NCT00274118) (Cummings, Lee, Ritter, & Zhong, 2018)	The data that support the findings of this study are openly available in PubMed at https://doi.org/10.1016/j.trci.2018.03.009 (Cummings et al., 2018)

Sources: clinicaltrials.gov and druginfo.nlm.nih.gov

## 5 CONCLUSION

To date, only a total of five FDA (Food and Drug Administration)‐approved drugs for treating AD are available, which indicates the level of complexity in addressing research problems among neurodegenerative diseases. This is partially because AD research in the last decade has mostly focused on conventional AD pathophysiology, which is not easily reversible. Moreover, it is evident that patients with T2DM are at a higher risk of developing AD symptoms, which is why the concept of metabolism‐dependent neurodegeneration mechanisms is gaining importance. This concept enables researchers to study neurodegenerative diseases such as AD with an entirely different perspective, through the lens of a metabolic disorder. The overlapping mechanisms of AD and T2DM justify why AD must be considered as “type 3 diabetes.” This fresh perspective takes us toward an entirely different approach, which involves targeting insulin signaling, and glucose metabolism as a novel therapeutic strategy for AD. A greater understanding of the underlying mechanisms of T2DM‐associated neurodegeneration will guide researchers to develop advanced AD management strategies. Thus, insulin sensitizers/insulin therapy and antidiabetic drugs are also among the latest focus of AD research.

In short, diabetes‐related neurodegeneration is a challenging problem, which needs to be explored further. The progress in AD research in this direction is considerable; however, much needs to be done in the near future. This fresh perspective opens the window to promising new developments in the treatment of several neurodegenerative diseases, especially Alzheimer's disease. These striking parallels are a matter of concern, not only for scientists but also for the public, because of the alarming increase in the number of patients with diabetes all over the world. Thus, by exploring new knowledge about the pathogenesis of diabetes‐associated neurodegeneration, we anticipate that scientists will develop more advanced and effective therapeutics in the near future.

## CONFLICT OF INTEREST

Authors declare no conflicts of interest.

## AUTHOR CONTRIBUTIONS

JM wrote the manuscript and contributed Figures 1 and 2. GS wrote the manuscript and contributed Figure 3. VD wrote the manuscript and had full access to all data in the study.

## Data Availability

The data that supports the findings of this study are available in the supplementary material of this article.

## References

[brb31577-bib-0001] Abdelalim, E. M. , Bonnefond, A. , Bennaceur‐Griscelli, A. , & Froguel, P. (2014). Pluripotent stem cells as a potential tool for disease modelling and cell therapy in diabetes. Stem Cell Reviews and Reports, 10(3), 327–337. 10.1007/s12015-014-9503-6 24577791

[brb31577-bib-0002] Adler, B. L. , Yarchoan, M. , Hwang, H. M. , Louneva, N. , Blair, J. A. , Palm, R. , … Casadesus, G. (2014). Neuroprotective effects of the amylin analogue pramlintide on Alzheimer’s disease pathogenesis and cognition. Neurobiology of Aging, 35(4), 793–801. 10.1016/j.neurobiolaging.2013.10.076 24239383

[brb31577-bib-0003] Akter, R. , Cao, P. , Noor, H. , Ridgway, Z. , Tu, L.‐H. , Wang, H. , … Raleigh, D. P. (2016). Islet amyloid polypeptide: structure, function, and pathophysiology. Journal of Diabetes Research, 2016, 1–18. 10.1155/2016/2798269 PMC4662979 26649319

[brb31577-bib-0004] Anderson, R. E. , Tan, W. K. , Martin, H. S. , & Meyer, F. B. (1999). Effects of glucose and PaO2 modulation on cortical intracellular acidosis, NADH redox state, and infarction in the ischemic penumbra. Stroke, 30(1), 160–170. 10.1161/01.STR.30.1.160 9880405

[brb31577-bib-0005] Ashok, A. , & Singh, N. (2018). Prion protein modulates glucose homeostasis by altering intracellular iron. Scientific Reports, 8(1), 6556 10.1038/s41598-018-24786-1 29700330 PMC5919926

[brb31577-bib-0006] Aviles‐Olmos, I. , Dickson, J. , Kefalopoulou, Z. , Djamshidian, A. , Ell, P. , Soderlund, T. , … Foltynie, T. (2013). Exenatide and the treatment of patients with Parkinson’s disease. Journal of Clinical Investigation, 123(6), 2730–2736. 10.1172/JCI68295 23728174 PMC3668846

[brb31577-bib-0007] Bae, C. S. , & Song, J. (2017). The role of glucagon‐like peptide 1 (GLP1) in type 3 diabetes: GLP‐1 controls insulin resistance, neuroinflammation and neurogenesis in the brain. International Journal of Molecular Sciences, 18(11), 2493 10.3390/ijms18112493 PMC5713459 29165354

[brb31577-bib-0008] Bahniwal, M. , Little, J. P. , & Klegeris, A. (2017). High glucose enhances neurotoxicity and inflammatory cytokine secretion by stimulated human astrocytes. Current Alzheimer Research, 14(7), 731–741. 10.2174/1567205014666170117104053 28124586

[brb31577-bib-0009] Batista, A. F. , Forny‐Germano, L. , Clarke, J. R. , Lyra e Silva, N. M. , Brito‐Moreira, J. , Boehnke, S. E. , … De Felice, F. G. (2018). The diabetes drug liraglutide reverses cognitive impairment in mice and attenuates insulin receptor and synaptic pathology in a non‐human primate model of Alzheimer’s disease. Journal of Pathology, 245(1), 85–100. 10.1002/path.5056 29435980 PMC5947670

[brb31577-bib-0010] Bernardes, R. , da Silva Filho, S. , Oliveira Barbosa, J. H. , Rondinoni, C. , dos Santos, A. C. , Garrido Salmon, C. E. , da Costa Lima, N. K. , … Moriguti, J. C. (2017). Neuro‐degeneration profile of Alzheimer’s patients: A brain morphometry study. NeuroImage: Clinical, 15, 15–24. 10.1016/j.nicl.2017.04.001 28459000 PMC5397580

[brb31577-bib-0011] Bharadwaj, P. , Wijesekara, N. , Liyanapathirana, M. , Newsholme, P. , Ittner, L. , Fraser, P. , & Verdile, G. (2017). The link between type 2 diabetes and neurodegeneration: Roles for amyloid‐β, amylin, and tau proteins. Journal of Alzheimer’s Disease, 59(2), 421–432. 10.3233/JAD-161192 28269785

[brb31577-bib-0012] Bianchi, M. , & Manco, M. (2018). Pin1 modulation in physiological status and neurodegeneration. Any contribution to the pathogenesis of type 3 diabetes? International Journal of Molecular Sciences, 19(8), 2319 10.3390/ijms19082319 PMC6121450 30096758

[brb31577-bib-0013] Blázquez, E. , Velázquez, E. , Hurtado-Carneiro, V. , & Ruiz-Albusac, J. M. (2014). Insulin in the brain: its pathophysiological implications for States related with central insulin resistance, type 2 diabetes and Alzheimer’s disease. Frontiers in Endocrinology, 5, 161 10.3389/fendo.2014.00161 25346723 PMC4191295

[brb31577-bib-0014] Boyd, F. T. , Clarke, D. W. , Muther, T. F. , & Raizada, M. K. (1985). Insulin receptors and insulin modulation of norepinephrine uptake in neuronal cultures from rat brain. Journal of Biological Chemistry, 60(29), 15880‐15884. 3905797

[brb31577-bib-0015] Calvo‐Ochoa, E. , & Arias, C. (2015). Cellular and metabolic alterations in the hippocampus caused by insulin signalling dysfunction and its association with cognitive impairment during aging and Alzheimer’s disease: Studies in animal models. Diabetes/Metabolism Research and Reviews, 31(1), 1–13. 10.1002/dmrr.2531 24464982

[brb31577-bib-0016] Chakrabarti, S. , Khemka, V. K. , Banerjee, A. , Chatterjee, G. , Ganguly, A. , & Biswas, A. (2015). Metabolic risk factors of sporadic Alzheimer’s disease: Implications in the pathology, pathogenesis and treatment. Aging and Disease, 6(4), 282–299. 10.14336/AD.2014.002 26236550 PMC4509477

[brb31577-bib-0017] Chapman, C. D. , Schiöth, H. B. , Grillo, C. A. , & Benedict, C. (2018). Intranasal insulin in Alzheimer’s disease: Food for thought. Neuropharmacology, 136, 196–201. 10.1016/j.neuropharm.2017.11.037 29180222 PMC10523803

[brb31577-bib-0018] Chen, J.‐L. , Luo, C. , Pu, D. , Zhang, G.‐Q. , Zhao, Y.‐X. , Sun, Y. , … Xiao, Q. (2019). Metformin attenuates diabetes‐induced tau hyperphosphorylation in vitro and in vivo by enhancing autophagic clearance. Experimental Neurology, 311, 44–56. 10.1016/j.expneurol.2018.09.008 30219731

[brb31577-bib-0019] Cheng, G. , Huang, C. , Deng, H. , & Wang, H. (2012). Diabetes as a risk factor for dementia and mild cognitive impairment: A meta‐analysis of longitudinal studies. Internal Medicine Journal, 42(5), 484–491. 10.1111/j.1445-5994.2012.02758.x 22372522

[brb31577-bib-0020] Craft, S. (2006). Insulin resistance syndrome and Alzheimer disease: Pathophysiologic mechanisms and therapeutic implications. Alzheimer Disease and Associated Disorders, 20(4), 298–301. 10.1097/01.wad.0000213866.86934.7e 17132977

[brb31577-bib-0021] Cummings, J. , Lee, G. , Ritter, A. , & Zhong, K. (2018). Alzheimer’s disease drug development pipeline: 2018. Alzheimer’s and Dementia: Translational Research and Clinical Interventions, 4, 195–214. 10.1016/j.trci.2018.03.009 29955663 PMC6021548

[brb31577-bib-0022] de la Monte, S. M. (2014). Type 3 diabetes is sporadic Alzheimer׳s disease: Mini‐review. European Neuropsychopharmacology, 24(12), 1954–1960. 10.1016/j.euroneuro.2014.06.008 25088942 PMC4444430

[brb31577-bib-0023] De La Monte, S. M. , & Wands, J. R. (2008). Alzheimer’s disease is type 3 diabetes‐evidence reviewed. Journal of Diabetes Science and Technology, 2(6), 1101–1113. 10.1177/193229680800200619 19885299 PMC2769828

[brb31577-bib-0024] Despa, F. , & DeCarli, C. (2013). Amylin: What might be its role in Alzheimer’s disease and how could this affect therapy? Expert Review of Proteomics, 10(5), 403–405. 10.1586/14789450.2013.841549 24117198 PMC4068803

[brb31577-bib-0025] Dosunmu, R. , Wu, J. , Basha, M. R. , & Zawia, N. H. (2007). Environmental and dietary risk factors in Alzheimer’s disease. Expert Review of Neurotherapeutics, 7(7), 887–900. 10.1586/14737175.7.7.887 17610395

[brb31577-bib-0026] Duarte, A. I. , Proença, T. , Oliveira, C. R. , Santos, M. S. , & Rego, A. C. (2006). Insulin restores metabolic function in cultured cortical neurons subjected to oxidative stress. Diabetes, 55(10), 2863–2870. 10.2337/db06-0030 17003354

[brb31577-bib-0027] Edvinsson, L. , Goadsby, P. J. , & Uddman, R. (2005). Amylin: localization, effects on cerebral arteries and on local cerebral blood flow in the cat. The Scientific World Journal, 1, 168–180. 10.1100/tsw.2001.23 PMC6084712 12805660

[brb31577-bib-0028] Femminella, G. D. , Frangou, E. , Love, S. B. , Busza, G. , Holmes, C. , Ritchie, C. , … Edison, P. (2019). Evaluating the effects of the novel GLP‐1 analogue liraglutide in Alzheimer’s disease: Study protocol for a randomised controlled trial (ELAD study). Trials, 20(1), 191 10.1186/s13063-019-3259-x 30944040 PMC6448216

[brb31577-bib-0029] Ferreira, S. T. , Clarke, J. R. , Bomfim, T. R. , & De Felice, F. G. (2014). Inflammation, defective insulin signaling, and neuronal dysfunction in Alzheimer’s disease. Alzheimer’s and Dementia, 10, S76–S83. 10.1016/j.jalz.2013.12.010 24529528

[brb31577-bib-0030] Frazier, H. N. , Ghoweri, A. O. , Anderson, K. L. , Lin, R. L. , Porter, N. M. , & Thibault, O. (2019). Broadening the definition of brain insulin resistance in aging and Alzheimer’s disease. Experimental Neurology, 313, 79–87. 10.1016/j.expneurol.2018.12.007 30576640 PMC6370304

[brb31577-bib-0031] Friedman, L. G. , Qureshi, Y. H. , & Yu, W. H. (2015). Promoting Autophagic Clearance: Viable Therapeutic Targets in Alzheimer’s Disease. Neurotherapeutics: the Journal of the American Society for Experimental NeuroTherapeutics, 12(1), 94–108. 10.1007/s13311-014-0320-z 25421002 PMC4322072

[brb31577-bib-0032] Galimberti, D. , & Scarpini, E. (2017). Pioglitazone for the treatment of Alzheimer’s disease. Expert Opinion on Investigational Drugs, 26(1), 97–101. 10.1080/13543784.2017.1265504 27885860

[brb31577-bib-0033] Gault, V. A. , & Hölscher, C. (2008a). GLP‐1 agonists facilitate hippocampal LTP and reverse the impairment of LTP induced by beta‐amyloid. European Journal of Pharmacology, 587(1‐3), 112–117. 10.1016/j.ejphar.2008.03.025 18466898

[brb31577-bib-0034] Gault, V. A. , & Hölscher, C. (2008b). Protease‐Resistant Glucose‐Dependent Insulinotropic Polypeptide Agonists Facilitate Hippocampal LTP and Reverse the Impairment of LTP Induced by Beta‐Amyloid. Journal of Neurophysiology, 99(4), 1590–1595. 10.1152/jn.01161.2007 18234983

[brb31577-bib-0035] Gault, V. A. , O’Harte, F. P. M. , & Flatt, P. R. (2003). Glucose‐dependent insulinotropic polypeptide (GIP): Anti‐diabetic and anti‐obesity potential? Neuropeptides, 37(5), 253–263. 10.1016/j.npep.2003.09.002 14607102

[brb31577-bib-0036] Geldmacher, D. S. , Fritsch, T. , McClendon, M. J. , & Landreth, G. (2011). A randomized pilot clinical trial of the safety of pioglitazone in treatment of patients with Alzheimer disease. Archives of Neurology, 68(1), 45–50. 10.1001/archneurol.2010.229 20837824

[brb31577-bib-0037] Griffith, C. M. , Eid, T. , Rose, G. M. , & Patrylo, P. R. (2018). Evidence for altered insulin receptor signaling in Alzheimer’s disease. Neuropharmacology, 136, 202–215. 10.1016/j.neuropharm.2018.01.008 29353052

[brb31577-bib-0038] Grizzanti, J. , Corrigan, R. , & Casadesus, G. (2018). Neuroprotective effects of amylin analogues on Alzheimer’s disease pathogenesis and cognition. Journal of Alzheimer’s Disease, 66(1), 11–23. 10.3233/JAD-180433 PMC6537587 30282360

[brb31577-bib-0039] Grizzanti, J. , Lee, H. G. , Camins, A. , Pallas, M. , & Casadesus, G. (2016). The therapeutic potential of metabolic hormones in the treatment of age‐related cognitive decline and Alzheimer’s disease. Nutrition Research, 36(12), 1305–1315. 10.1016/j.nutres.2016.11.002 27923524 PMC5490446

[brb31577-bib-0040] Gudala, K. , Bansal, D. , Schifano, F. , & Bhansali, A. (2013). Diabetes mellitus and risk of dementia: A meta‐analysis of prospective observational studies. Journal of Diabetes Investigation, 4(6), 640–650. 10.1111/jdi.12087 24843720 PMC4020261

[brb31577-bib-0041] Hannah, J. A. (1936). A case of Alzheimer’s disease with neuropathological findings. Canadian Medical Association Journal, 35(4), 361–366. 20320397 PMC1561804

[brb31577-bib-0042] Harding, J. L. , Pavkov, M. E. , Magliano, D. J. , Shaw, J. E. , & Gregg, E. W. (2019). Global trends in diabetes complications: A review of current evidence. Diabetologia, 62(1), 3–16. 10.1007/s00125-018-4711-2 30171279

[brb31577-bib-0043] Hascup, E. R. , Broderick, S. O. , Russell, M. K. , Fang, Y. , Bartke, A. , Boger, H. A. , & Hascup, K. N. (2019). Diet‐induced insulin resistance elevates hippocampal glutamate as well as VGLUT1 and GFAP expression in AβPP/PS1 mice. Journal of Neurochemistry, 148(2), 219–237. 10.1111/jnc.14634 30472734 PMC6438176

[brb31577-bib-0044] Hsu, C. C. , Wahlqvist, M. L. , Lee, M. S. , & Tsai, H. N. (2011). Incidence of dementia is increased in type 2 diabetes and reduced by the use of sulfonylureas and metformin. Journal of Alzheimer’s Disease, 24(3), 485–493. 10.3233/JAD-2011-101524 21297276

[brb31577-bib-0045] Huang, N.‐Q. , Jin, H. , Zhou, S.‐Y. , Shi, J.‐S. , & Jin, F. (2017). TLR4 is a link between diabetes and Alzheimer’s disease. Behavioural Brain Research, 316, 234–244. 10.1016/j.bbr.2016.08.047 27591966

[brb31577-bib-0046] Iqbal, K. , Liu, F. , Gong, C.‐X. , Alonso, A. D. C. , & Grundke‐Iqbal, I. (2009). Mechanisms of tau‐induced neurodegeneration. Acta Neuropathologica, 118(1), 53–69. 10.1007/s00401-009-0486-3 19184068 PMC2872491

[brb31577-bib-0047] Irwin, N. , Green, B. D. , Gault, V. A. , Harriot, P. , O’Harte, F. P. M. , & Flatt, P. R. (2006). Stable agonist of glucose‐dependent insulinotropic polypeptide (GIP) restores pancreatic beta cell glucose responsiveness but not glucose intolerance in aging mice. Experimental Gerontology, 41(2), 151–156. 10.1016/j.exger.2005.11.006 16378704

[brb31577-bib-0048] Ito, F. , Sono, Y. , & Ito, T. (2019). Measurement and Clinical Significance of Lipid Peroxidation as a Biomarker of Oxidative Stress: Oxidative Stress in Diabetes, Atherosclerosis, and Chronic Inflammation. Antioxidants, 8(3), 72 10.3390/antiox8030072 PMC6466575 30934586

[brb31577-bib-0049] Jackson, K. , Barisone, G. A. , Diaz, E. , Jin, L. W. , DeCarli, C. , & Despa, F. (2013). Amylin deposition in the brain: A second amyloid in Alzheimer disease? Annals of Neurology, 74(4), 517–526. 10.1002/ana.23956 23794448 PMC3818462

[brb31577-bib-0050] Jarosz‐Griffiths, H. H. , Noble, E. , Rushworth, J. V. , & Hooper, N. M. (2016). Amyloid‐β receptors: The good, the bad, and the prion protein. Journal of Biological Chemistry, 291(7), 3174–3183. 10.1074/jbc.R115.702704 26719327 PMC4751366

[brb31577-bib-0051] Jayaraman, A. , & Pike, C. J. (2014). Alzheimer’s disease and type 2 diabetes: Multiple mechanisms contribute to interactions. Current Diabetes Reports, 14(4), 476 10.1007/s11892-014-0476-2 24526623 PMC3985543

[brb31577-bib-0052] Jhamandas, J. H. , Li, Z. , Westaway, D. , Yang, J. , Jassar, S. , & MacTavish, D. (2011). Actions of β‐amyloid protein on human neurons are expressed through the amylin receptor. American Journal of Pathology, 178(1), 140–149. 10.1016/j.ajpath.2010.11.022 21224052 PMC3070588

[brb31577-bib-0053] Johnson, L. A. , Torres, E. R. S. , Impey, S. , Stevens, J. F. , & Raber, J. (2017). Apolipoprotein E4 and insulin resistance interact to impair cognition and alter the epigenome and metabolome. Scientific Reports, 7(1), 43701 10.1038/srep43701 28272510 PMC5341123

[brb31577-bib-0054] Kalaria, R. N. , Maestre, G. E. , Arizaga, R. , Friedland, R. P. , Galasko, D. , Hall, K. , … World Federation of Neurology Dementia Research Group (2008). Alzheimer's disease and vascular dementia in developing countries: prevalence, management, and risk factors. The Lancet Neurology, 7(9), 812–826. 10.1016/S1474-4422(08)70169-8 18667359 PMC2860610

[brb31577-bib-0055] Kamada, H. , Yu, F. , Nito, C. , & Chan, P. H. (2007). Influence of hyperglycemia on oxidative stress and matrix metalloproteinase‐9 activation after focal cerebral ischemia/reperfusion in rats: Relation to blood‐brain barrier dysfunction. Stroke, 38(3), 1044–1049. 10.1161/01.STR.0000258041.75739.cb 17272778 PMC1828129

[brb31577-bib-0056] Kanamori, H. , Takemura, G. , Goto, K. , Tsujimoto, A. , Mikami, A. , Ogino, A. , … Minatoguchi, S. (2015). Autophagic adaptations in diabetic cardiomyopathy differ between type 1 and type 2 diabetes. Autophagy, 11(7), 1146–1160. 10.1080/15548627.2015.1051295 26042865 PMC4590644

[brb31577-bib-0057] Kaneto, H. , Katakami, N. , Matsuhisa, M. , & Matsuoka, T. (2010). Role of reactive oxygen species in the progression of type 2 diabetes and atherosclerosis. Mediators of Inflammation, 2010, 1–11. 10.1155/2010/453892 PMC2825658 20182627

[brb31577-bib-0058] Kim, B. , & Feldman, E. L. (2015). Insulin resistance as a key link for the increased risk of cognitive impairment in the metabolic syndrome. Experimental & Molecular Medicine, 47(3), e149–e149. 10.1038/emm.2015.3 25766618 PMC4351418

[brb31577-bib-0059] Kiriyama, Y. , & Nochi, H. (2015). The function of autophagy in neurodegenerative diseases. International Journal of Molecular Sciences, 16(11), 26797–26812. 10.3390/ijms161125990 26569220 PMC4661849

[brb31577-bib-0060] Komatsu, M. , Waguri, S. , Chiba, T. , Murata, S. , Iwata, J.‐I. , Tanida, I. , … Tanaka, K. (2006). Loss of autophagy in the central nervous system causes neurodegeneration in mice. Nature, 441(7095), 880–884. 10.1038/nature04723 16625205

[brb31577-bib-0061] Kulas, J. A. , Franklin, W. F. , Smith, N. A. , Manocha, G. D. , Puig, K. L. , Nagamoto‐Combs, K. , … Combs, C. K. (2018). Ablation of amyloid precursor protein increases insulin‐degrading enzyme levels and activity in brain and peripheral tissues. American Journal of Physiology‐Endocrinology and Metabolism, 316(1), E106–E120. 10.1152/ajpendo.00279.2018 30422705 PMC6417684

[brb31577-bib-0062] Lim, Y. A. , Ittner, L. M. , Lim, Y. L. , & Götz, J. (2008). Human but not rat amylin shares neurotoxic properties with Aβ42 in long‐term hippocampal and cortical cultures. FEBS Letters, 582(15), 2188–2194. 10.1016/j.febslet.2008.05.006 18486611

[brb31577-bib-0063] Lim, Y. A. , Rhein, V. , Baysang, G. , Meier, F. , Poljak, A. , Raftery, M. J. , … Götz, J. (2010). Aβ and human amylin share a common toxicity pathway via mitochondrial dysfunction. Proteomics, 10(8):1621–1633. 10.1002/pmic.200900651 20186753

[brb31577-bib-0064] Liu, Z. , Li, T. , Li, P. , Wei, N. , Zhao, Z. , Liang, H. , … Wei, J. (2015). The ambiguous relationship of oxidative stress, tau hyperphosphorylation, and autophagy dysfunction in Alzheimer’s disease. Oxidative Medicine and Cellular Longevity, 2015, 1–12. 10.1155/2015/352723 PMC4485995 26171115

[brb31577-bib-0065] Luchsinger, J. A. , Tang, M.‐X. , Shea, S. , & Mayeux, R. (2004). Hyperinsulinemia and risk of Alzheimer disease. Neurology, 63(7), 1187–1192. 10.1212/01.wnl.0000140292.04932.87 15477536

[brb31577-bib-0066] Lutz, T. A. , & Meyer, U. (2015). Amylin at the interface between metabolic and neurodegenerative disorders. Frontiers in Neuroscience, 9, 216 10.3389/fnins.2015.00216 26136651 PMC4468610

[brb31577-bib-0067] McClean, P. L. , & Hölscher, C. (2014a). Liraglutide can reverse memory impairment, synaptic loss and reduce plaque load in aged APP/PS1 mice, a model of Alzheimer’s disease. Neuropharmacology, 76, 57–67. 10.1016/j.neuropharm.2013.08.005 23973293

[brb31577-bib-0068] McClean, P. L. , & Hölscher, C. (2014b). Lixisenatide, a drug developed to treat type 2 diabetes, shows neuroprotective effects in a mouse model of Alzheimer’s disease. Neuropharmacology, 86, 241–258. 10.1016/j.neuropharm.2014.07.015 25107586

[brb31577-bib-0069] Mehla, J. , Chauhan, B. C. , & Chauhan, N. B. (2014). Experimental induction of type 2 diabetes in aging‐accelerated mice triggered alzheimer‐like pathology and memory deficits. Journal of Alzheimer’s Disease, 39(1), 145–162. 10.3233/JAD-131238 PMC3941701 24121970

[brb31577-bib-0070] Merelli, A. , Rodríguez, J. C. G. , Folch, J. , Regueiro, M. R. , Camins, A. , & Lazarowski, A. (2018). Understanding the role of hypoxia inducible factor during neurodegeneration for new therapeutics opportunities. Current Neuropharmacology, 16(10), 1484–1498. 10.2174/1570159X16666180110130253 29318974 PMC6295932

[brb31577-bib-0071] Mietelska‐Porowska, A. , Wasik, U. , Goras, M. , Filipek, A. , & Niewiadomska, G. (2014). Tau protein modifications and interactions: Their role in function and dysfunction. International Journal of Molecular Sciences, 15(3), 4671–4713. 10.3390/ijms15034671 24646911 PMC3975420

[brb31577-bib-0072] Mietlicki‐Baase, E. G. (2016). Amylin‐mediated control of glycemia, energy balance, and cognition. Physiology and Behavior, 162, 130–140. 10.1016/j.physbeh.2016.02.034 26922873 PMC4899204

[brb31577-bib-0073] Mietlicki‐Baase, E. G. , & Hayes, M. R. (2014). Amylin activates distributed CNS nuclei to control energy balance. Physiology and Behavior, 136, 39–46. 10.1016/j.physbeh.2014.01.013 24480072 PMC4113606

[brb31577-bib-0074] Mitsukawa, T. , Takemura, J. , Asai, J. , Nakazato, M. , Kangawa, K. , Matsuo, H. , & Matsukura, S. (1990). Islet amyloid polypeptide response to glucose, insulin, and somatostatin analogue administration. Diabetes, 39(5), 639–642. 10.2337/diab.39.5.639 1970540

[brb31577-bib-0075] Moon, H. S. , Chamberland, J. P. , & Mantzoros, C. S. (2012). Amylin and leptin activate overlapping signalling pathways in an additive manner in mouse GT1‐7 hypothalamic, C 2C 12 muscle and AML12 liver cell lines. Diabetologia, 55(1), 215–225. 10.1007/s00125-011-2332-0 21997794 PMC3780406

[brb31577-bib-0076] Moreira, R. O. , Campos, S. C. , & Soldera, A. L. (2013). Type 2 diabetes mellitus and Alzheimer’s disease: From physiopathology to treatment implications. Diabetes/Metabolism Research and Reviews. 10.1002/dmrr.2442 23868462

[brb31577-bib-0077] Moroz, N. , Tong, M. , Longato, L. , Xu, H. , & De La Monte, S. M. (2008). Limited Alzheimer-type neurodegeneration in experimental obesity and type 2 diabetes mellitus. Journal of Alzheimer’s Disease, 10.3233/JAD-2008-15103 PMC10074393 18780965

[brb31577-bib-0078] Nagamatsu, S. , Nishi, M. , & Steiner, D. F. (1991). Biosynthesis of islet amyloid polypeptide: Elevated expression in mouse βTC3 cells. Journal of Biological Chemistry, 266, 13737–13741. 1856207

[brb31577-bib-0079] Nasoohi, S. , Parveen, K. , & Ishrat, T. (2018). Metabolic syndrome, brain insulin resistance, and Alzheimer’s disease: Thioredoxin Interacting Protein (TXNIP) and inflammasome as core amplifiers. Journal of Alzheimer’s Disease, 66(3), 857–885. 10.3233/JAD-180735 30372683

[brb31577-bib-0080] Nassar, S. Z. , Badae, N. M. , & Issa, Y. A. (2018). Effect of amylin on memory and central insulin resistance in a rat model of Alzheimer’s disease. Archives of Physiology and Biochemistry. 10.1080/13813455.2018.1534244 30449203

[brb31577-bib-0081] NCD Risk factor Collaboration (NCD‐RisC) (2016). Worldwide trends in diabetes since 1980: A pooled analysis of 751 population‐based studies with 4·4 million participants. The Lancet, 387(10027), 1513–1530. 10.1016/S0140-6736(16)00618-8 PMC5081106 27061677

[brb31577-bib-0082] Ng, T. P. , Feng, L. , Yap, K. B. , Lee, T. S. , Tan, C. H. , & Winblad, B. (2014). Long‐term metformin usage and cognitive function among older adults with diabetes. Journal of Alzheimer’s Disease, 41(1), 61–68. 10.3233/JAD-131901 24577463

[brb31577-bib-0083] Nguyen, T. T. L. , Chan, L. C. , Borreginne, K. , Kale, R. P. , Hu, C. , & Tye, S. J. (2018). A review of brain insulin signaling in mood disorders: From biomarker to clinical target. Neuroscience and Biobehavioral Reviews, 92, 7–15. 10.1016/j.neubiorev.2018.05.014 29758232

[brb31577-bib-0084] O’Brien, R. J. , & Wong, P. C. (2011). Amyloid precursor protein processing and Alzheimer's disease. Annual Review of Neuroscience, 34(1), 185–204. 10.1146/annurev-neuro-061010-113613 PMC3174086 21456963

[brb31577-bib-0085] Oliveira, W. H. , Nunes, A. K. , França, M. E. R. , Santos, L. A. , Lós, D. B. , Rocha, S. W. , … Peixoto, C. A. (2016). Effects of metformin on inflammation and short‐term memory in streptozotocin‐induced diabetic mice. Brain Research, 1644, 149–160. 10.1016/j.brainres.2016.05.013 27174003

[brb31577-bib-0086] Patrick, S. , Corrigan, R. , Grizzanti, J. , Mey, M. , Blair, J. , Pallas, M. , … Casadesus, G. (2019). Neuroprotective effects of the amylin analog, pramlintide, on Alzheimer’s disease are associated with oxidative stress regulation mechanisms. Journal of Alzheimer’s Disease, 69(1), 157–168. 10.3233/JAD-180421 PMC6584632 30958347

[brb31577-bib-0087] Pearson, H. A. , & Peers, C. (2006). Physiological roles for amyloid beta peptides. The Journal of Physiology, 575(Pt 1), 5–10. 10.1113/jphysiol.2006.111203 16809372 PMC1819417

[brb31577-bib-0088] Pintana, H. , Apaijai, N. , Pratchayasakul, W. , Chattipakorn, N. , & Chattipakorn, S. C. (2012). Effects of metformin on learning and memory behaviors and brain mitochondrial functions in high fat diet induced insulin resistant rats. Life Sciences, 91(11‐12), 409–414. 10.1016/j.lfs.2012.08.017 22925597

[brb31577-bib-0089] Plum, L. , Schubert, M. , & Brüning, J. C. (2005). The role of insulin receptor signaling in the brain. Trends in Endocrinology and Metabolism, 16(2), 59–65. 10.1016/j.tem.2005.01.008 15734146

[brb31577-bib-0090] Pugazhenthi, S. , Qin, L. , & Reddy, P. H. (2017). Common neurodegenerative pathways in obesity, diabetes, and Alzheimer's disease. Biochimica Et Biophysica Acta (BBA) ‐ Molecular Basis of Disease, 1863(5), 1037–1045. 10.1016/j.bbadis.2016.04.017 27156888 PMC5344771

[brb31577-bib-0091] Qin, L. , Chong, T. , Rodriguez, R. , & Pugazhenthi, S. (2016). Glucagon‐like peptide‐1‐mediated modulation of inflammatory pathways in the diabetic brain: relevance to Alzheimer’s disease. Current Alzheimer Research, 13(12), 1346–1355. 10.2174/1567205013666160401114751 27033055

[brb31577-bib-0092] Ropper, A. H. (1979). A rational approach to dementia. Canadian Medical Association Journal, 121(9), 1175. 159118 PMC1704685

[brb31577-bib-0093] Roth, J. D. (2013). Amylin and the regulation of appetite and adiposity: Recent advances in receptor signaling, neurobiology and pharmacology. Current Opinion in Endocrinology, Diabetes and Obesity, 20(1), 8–13. 10.1097/MED.0b013e32835b896f 23183359

[brb31577-bib-0094] Sanke, T. , Bell, G. I. , Sample, C. , Rubenstein, A. H. , & Steiner, D. F. (1988). An islet amyloid peptide is derived from an 89-amino acid precursor by proteolytic processing. The Journal of Biological Chemistry, 263(33), 17243–17246. Retrieved from http://www.ncbi.nlm.nih.gov/pubmed/3053705 3053705

[brb31577-bib-0095] Sagare, A. P. , Sweeney, M. D. , Nelson, A. R. , Zhao, Z. , & Zlokovic, B. V. (2019). Prion protein antagonists rescue Alzheimer’s amyloid‐β‐related cognitive deficits. Trends in Molecular Medicine, 25(2), 74–76. 10.1016/j.molmed.2019.01.001 30661727 PMC6377285

[brb31577-bib-0096] Saini, V. (2010). Molecular mechanisms of insulin resistance in type 2 diabetes mellitus. World Journal of Diabetes, 1(3), 68 10.4239/wjd.v1.i3.68 21537430 PMC3083885

[brb31577-bib-0097] Seaquist, E. R. (2010). The final frontier: How does diabetes affect the brain? Diabetes, 59(1), 4–5. 10.2337/db09-1600 20040482 PMC2797942

[brb31577-bib-0098] Shinohara, M. , & Sato, N. (2017). Bidirectional interactions between diabetes and Alzheimer’s disease. Neurochemistry International, 108, 296–302. 10.1016/J.NEUINT.2017.04.020 28551028

[brb31577-bib-0099] Simcox, J. A. , & McClain, D. A. (2013). Iron and diabetes risk. Cell Metabolism, 17(3), 329–341. 10.1016/j.cmet.2013.02.007 23473030 PMC3648340

[brb31577-bib-0100] Skeberdis, V. A. , Lan, J.‐Y. , Zheng, X. , Zukin, R. S. , & Bennett, M. V. L. (2002). Insulin promotes rapid delivery of N‐methyl‐D‐ aspartate receptors to the cell surface by exocytosis. Proceedings of the National Academy of Sciences, 98(6), 3561–3566. 10.1073/pnas.051634698 PMC30692 11248117

[brb31577-bib-0101] Sripetchwandee, J. , Chattipakorn, N. , & Chattipakorn, S. C. (2018). Links between obesity‐induced brain insulin resistance, brain mitochondrial dysfunction, and dementia. Frontiers in Endocrinology, 9, 496 10.3389/fendo.2018.00496 30233495 PMC6127253

[brb31577-bib-0102] Stockhorst, U. , De Fries, D. , Steingrueber, H. J. , & Scherbaum, W. A. (2004). Insulin and the CNS: Effects on food intake, memory, and endocrine parameters and the role of intranasal insulin administration in humans. Physiology and Behavior, 83(1), 47–54. 10.1016/j.physbeh.2004.07.022 15501490

[brb31577-bib-0103] Study to Evaluate the Effect of Lixisenatide in Patient With Parkinson’s Disease (n.d.). Tabular View ‐ ClinicalTrials.gov. Retrieved from https://clinicaltrials.gov/ct2/show/record/NCT03439943

[brb31577-bib-0104] Tai, J. , Liu, W. , Li, Y. , Li, L. , & Hölscher, C. (2018). Neuroprotective effects of a triple GLP‐1/GIP/glucagon receptor agonist in the APP/PS1 transgenic mouse model of Alzheimer’s disease. Brain Research, 1678, 64–74. 10.1016/j.brainres.2017.10.012 29050859

[brb31577-bib-0105] Tomlinson, D. R. , & Gardiner, N. J. (2008). Glucose neurotoxicity. Nature Reviews Neuroscience, 9(1), 36–45. 10.1038/nrn2294 18094705

[brb31577-bib-0106] Trevaskis, J. L. , Turek, V. F. , Wittmer, C. , Griffin, P. S. , Wilson, J. K. , Reynolds, J. M. , … Roth, J. D. (2010). Enhanced amylin‐mediated body weight loss in estradiol‐deficient diet‐induced obese rats. Endocrinology, 151(12), 5657–5668. 10.1210/en.2010-0590 20962049

[brb31577-bib-0107] Tumminia, A. , Vinciguerra, F. , Parisi, M. , & Frittitta, L. (2018). Type 2 diabetes mellitus and Alzheimer’s disease: Role of insulin signalling and therapeutic implications. International Journal of Molecular Sciences, 19(11), 3306– 10.3390/ijms19113306 PMC6275025 30355995

[brb31577-bib-0108] Van Der Heide, L. P. , Kamal, A. , Artola, A. , Gispen, W. H. , & Ramakers, G. M. J. (2005). Insulin modulates hippocampal activity‐dependent synaptic plasticity in a N‐methyl‐D‐aspartate receptor and phosphatidyl‐inositol‐3‐kinase‐dependent manner. Journal of Neurochemistry, 94(4), 1158–1166. 10.1111/j.1471-4159.2005.03269.x 16092951

[brb31577-bib-0109] Van Dyken, P. , & Lacoste, B. (2018). Impact of metabolic syndrome on neuroinflammation and the blood‐brain barrier. Frontiers in Neuroscience, 12, 930 10.3389/fnins.2018.00930 30618559 PMC6297847

[brb31577-bib-0110] Verma, N. , Ly, H. , Liu, M. , Chen, J. , Zhu, H. , Chow, M. , … Despa, F. (2016). Intraneuronal amylin deposition, peroxidative membrane injury and increased IL‐1β synthesis in brains of Alzheimer’s disease patients with type‐2 diabetes and in diabetic HIP rats. Journal of Alzheimer’s Disease, 53(1), 259–272. 10.3233/JAD-160047 PMC4920720 27163815

[brb31577-bib-0111] Walter, P. B. , Knutson, M. D. , Paler‐Martinez, A. , Lee, S. , Xu, Y. , Viteri, F. E. , & Ames, B. N. (2002). Iron deficiency and iron excess damage mitochondria and mitochondrial DNA in rats. Proceedings of the National Academy of Sciences, 99(4), 2264–2269. 10.1073/pnas.261708798 PMC122353 11854522

[brb31577-bib-0112] Wan, Q. , Xiong, Z. G. , Man, H. Y. , Ackerley, C. A. , Braunton, J. , Lu, W. Y. , Wang, Y. T. (1997). Recruitment of functional GABAA receptors to postsynaptic domains by insulin. Nature, 388(6643), 686–690. 10.1038/41792 9262404

[brb31577-bib-0113] Wang, E. , Zhu, H. , Wang, X. , Gower, A. C. , Wallack, M. , Blusztajn, J. K. , … Qiu, W. Q. (2017). Amylin treatment reduces neuroinflammation and ameliorates abnormal patterns of gene expression in the cerebral cortex of an Alzheimer’s disease mouse model. Journal of Alzheimer’s Disease, 56(1), 47–61. 10.3233/JAD-160677 PMC5331853 27911303

[brb31577-bib-0114] Wang, Q. , Yuan, J. , Yu, Z. , Lin, L. I. , Jiang, Y. , Cao, Z. , … Wang, X. (2018). FGF21 attenuates high‐fat diet‐induced cognitive impairment via metabolic regulation and anti‐inflammation of obese mice. Molecular Neurobiology, 55(6), 4702–4717. 10.1007/s12035-017-0663-7 28712011 PMC5971086

[brb31577-bib-0115] Wild, S. , Roglic, G. , Green, A. , Sicree, R. , & King, H. (2004). Global prevalence of diabetes. Diabetes Care, 27(5), 1047–1053. 10.2337/diacare.27.5.1047 15111519

[brb31577-bib-0116] Wilson, C. , Magnaudeix, A. , Yardin, C. , & Terro, F. (2014). Autophagy dysfunction and its link to Alzheimer’s disease and type II diabetes mellitus. CNS & Neurological Disorders ‐ Drug Targets, 13(2), 226–246. 10.2174/18715273113126660146 24059314

[brb31577-bib-0117] Ye, F. , Luo, Y. J. , Xiao, J. , Yu, N. W. , & Yi, G. (2016). Impact of insulin sensitizers on the incidence of dementia: A meta‐analysis. Dementia and Geriatric Cognitive Disorders, 41(5‐6), 251–260. 10.1159/000445941 27250528

[brb31577-bib-0118] Yu, Y. , Li, X. , Blanchard, J. , Li, Y. , Iqbal, K. , Liu, F. , & Gong, C. X. (2015). Insulin sensitizers improve learning and attenuate tau hyperphosphorylation and neuroinflammation in 3xTg‐AD mice. Journal of Neural Transmission, 122(4), 593–606. 10.1007/s00702-014-1294-z 25113171

[brb31577-bib-0119] Zhao, W. Q. , Chen, H. , Quon, M. J. , & Alkon, D. L. (2004). Insulin and the insulin receptor in experimental models of learning and memory. European Journal of Pharmacology, 490(1‐3), 71–81. 10.1016/j.ejphar.2004.02.045 15094074

